# An Inverse Agonist of Estrogen-Related Receptor Gamma, GSK5182, Enhances Na^+^/I^−^ Symporter Function in Radioiodine-Refractory Papillary Thyroid Cancer Cells

**DOI:** 10.3390/cells12030470

**Published:** 2023-02-01

**Authors:** Thoudam Debraj Singh, Jae Eon Lee, Kwang Hee Son, Bo Ra Lee, Sang Kyoon Kim, Deepak Gulwani, Vijaya Sarangthem, Yong Hyun Jeon

**Affiliations:** 1 Department of Medical Oncology Lab, All India Institute of Medical Sciences (AIIMS), New Delhi 110029, India; 2 Preclincial Research Center (PRL), Daegu-Gyeongbuk Medical Innovation Foundation (K-MEDI hub), Daegu 41061, Republic of Korea; 3 Department of Pathology, All India Institute of Medical Sciences (AIIMS), New Delhi 110029, India

**Keywords:** estrogen-related receptor gamma, radioiodine-refractory papillary thyroid cancer cells, sodium iodide symporter, radioiodine uptake, radioiodine therapy

## Abstract

Previously, we reported that an inverse agonist of estrogen-related receptor gamma (ERRγ), GSK5182, enhances sodium iodide (Na^+^/I^−^) symporter (NIS) function through mitogen-activated protein (MAP) kinase signaling in anaplastic thyroid cancer cells. This finding helped us to further investigate the effects of GSK5182 on NIS function in papillary thyroid cancer (PTC) refractory to radioactive iodine (RAI) therapy. Herein, we report the effects of ERRγ on the regulation of NIS function in RAI-resistant PTC cells using GSK5182. RAI-refractory BCPAP cells were treated with GK5182 for 24 h at various concentrations, and radioiodine avidity was determined with or without potassium perchlorate (KClO_4_) as an NIS inhibitor. We explored the effects of GSK5182 on ERRγ, the mitogen-activated protein (MAP) kinase pathway, and iodide metabolism-related genes. We examined whether the MAP pathway affected GSK5182-mediated NIS function using U0126, a selective MEK inhibitor. A clonogenic assay was performed to evaluate the cytotoxic effects of I-131. GSK5182 induced an increase in radioiodine avidity in a dose-dependent manner, and the enhanced uptake was completely inhibited by KClO_4_ in BCPAP cells. We found that ERRγ was downregulated and phosphorylated extracellular signal-regulated kinase (ERK)1/2 was upregulated in BCPAP cells, with an increase in total and membranous NIS and iodide metabolism-related genes. MEK inhibitors reversed the increase in radioiodine avidity induced by GSK5182. Clonogenic examination revealed the lowest survival in cells treated with a combination of GSK5182 and I-131 compared to those treated with either GSK518 or I-131 alone. We demonstrate that an inverse agonist of ERRγ, GSK5182, enhances the function of NIS protein via the modulation of ERRγ and MAP kinase signaling, thereby leading to increased responsiveness to radioiodine in RAI-refractory papillary thyroid cancer cells.

## 1. Introduction

The sodium iodide (Na^+^/I^−^) symporter (NIS) is a plasma membrane glycoprotein with 13 transmembrane domains that mediate the active influx of iodide into cells [1]. Endogenous NIS expression in normal thyroid and cancerous cells can be used as a diagnostic marker and therapeutic tool, owing to its capacity to facilitate iodide uptake. NIS-mediated radioactive iodine (RAI) has been proven to be an effective therapeutic method for eliminating malignant cells with minimal adverse effects. Most papillary thyroid cancers (PTCs) are effectively treated with RAI owing to the high expression of NIS and iodide metabolism-related genes (*TPO*, *TG*, and *TSHR*), and effective NIS function. However, PTCs often exhibit advanced de-differentiation that leads to a decrease in the levels of NIS and iodide metabolism-related genes [2,3], which impairs the ability to accumulate a high iodine concentration and hence their resistance to radionuclide therapy, which finally leads to poor prognosis. Many attempts have been made to explore the reason for the decrease in NIS expression and its function in poorly differentiated PTCs by modifying the epigenome [4,5]. Additionally, there have been attempts to restore NIS function in poorly differentiated PTC cells via gene delivery [6], treatment with chemicals or agents [7,8], and modulation of TSHR/cAMP/CREB/PAX8 [9], BRD4 [10], or mTORC1 [11] pathways. However, the outcomes of these endeavors have been unsatisfactory.

Estrogen-related receptors (ERRs) are closely related to estrogen receptors and share high levels of homology sequence identity but do not bind to estrogen [12]. The ERR subfamily consists of three members: ERRα, ERRβ, and ERRγ, which bind to classic estrogen response elements as either monomers or dimers. ERRs are predominantly expressed in most vital organs, including the liver, brain, kidneys, and pancreas [13]. Recent studies have shown that ERRγ plays an important role in vascular calcification [14] and bile acid metabolism in the liver [15] and is involved in several metabolic diseases, such as type 2 diabetes mellitus, alcohol-induced oxidative stress, liver injury, and microbial infection through impaired hepatic gluconeogenesis [16,17], hepatic insulin signaling [18], and iron metabolism [19]. Recently, we showed the important role of ERRγ in upregulating NIS function in anaplastic thyroid cancer (ATCs) using the selective inverse agonist of ERRγ, GSK5182 (a 4-hydroxy tamoxifen analog) [20]. Specifically, even though the detailed mechanism was not fully explored, we found that GSK5182 induced downregulation of endogenous ERRγ expression and subsequently activated the MAPK pathway, thereby leading to an increase in radioiodine avidity and restoration of RAI responsiveness in ATC cells. RAI-refractory PTC has a much larger patient population than ATC. Hence, it is necessary to investigate the effects of ERRγ regulation in the restoration of NIS expression and radioiodine avidity in RAI-refractory advanced PTCs, thereby leading to further expansion of our previous findings.

Herein, we attempted to demonstrate the effect of functional modulation of ERRγ on the restoration of NIS and iodide metabolism-related genes, radioiodine avidity, and responsiveness of RAI in ^131^I-resistant PTC cells.

## 2. Materials and Methods

### 2.1. Cells, Chemicals, and Antibodies

The human PTC cell line, BCPAP cells, was purchased from Deutsche Sammlung von Mikroorganismen und Zellkulturen. BCPAP cell lines were maintained in DMEM high supplemented with 10% FBS and 1% antibiotic-antimycotic (Hyclone) at 37 °C in a 5% CO_2_ atmosphere.

Primary mouse monoclonal human NIS-specific antibody (Thermo Scientific, Waltham, IL, USA), ERRγ (R&D, Minneapolis, MN, USA), hosphor-MAPK-p42/p44 (Cell Signaling, Danvers, MA, USA), GLUT-4 and β-actin antibodies (Abcam, MA, USA), thyroidperoxidase (TPO), thyroid stimulating hormone receptor (TSHR), thyroglobulin (TG), PAX-8, GLUT-1, and Na^+^K^+^-ATPase (Santa Cruz biotechnology Inc., Dallas, TX, USA) were purchased for the experiment.

### 2.2. Radioiodine Uptake Reporter Gene Assay

Detailed information on the radioiodine uptake assay has been provided in previous reports [20] and the supporting information.

### 2.3. F18-FDG Uptake Assay

Detailed information on the ^18^F-FDG Uptake assay has been provided in previous reports [20] and the supporting information.

### 2.4. Clonogenic Assay

Detailed information on the clonogenic assay has been provided in previous reports [20] and the supporting information.

### 2.5. Western Blot

Detailed information on western blot examination has been provided in previous reports [20] and the supporting information. Used antibodies was summarized in Table 1.

### 2.6. Quantitative RT-PCR

Detailed information on quantitative RT-PCR has been provided in previous reports [20] and the supporting information.

### 2.7. Statistical Analysis

All data are expressed as the mean ± standard deviation (SD) from at least three representative experiments. Statistical significance was determined using an unpaired Student’s *t*-test. *p* values of <0.05 were considered statistically significant.

## 3. Results

### 3.1. GSK5182 Enhanced Radioiodine Uptake in RAI-Refractory PTC Cells

**Figure 1:**
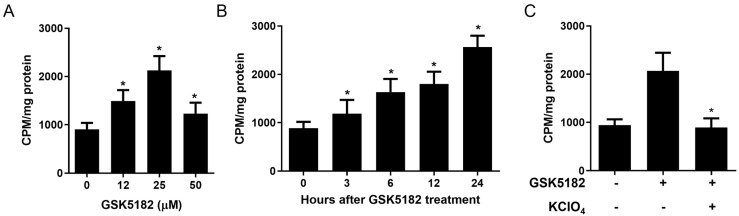
Radioiodine uptake in BCPAP cells by GSK5182. (**A**) Change in radioiodine uptake of BCPAP cells at different concentrations of GSK5182. (**B**) Time-dependent change in radioiodine uptake in GSK5182-treated BCPAP cells (GSK5182 = 25 µM). (**C**) Inhibition of radioiodine uptake in GSK5182-treated BCPAP cells by KClO_4_. BCPAP cells were treated with GSK5182 (25 µM) for 24 h. In case of the inhibition assay, GSK5182-treated cells were pre-treated with 0.3M KClO_4_ for 30 min and the radioiodine uptake assay was performed. *, *p* < 0.05. Data are the mean ± SD of three samples per group.

GSK5182 significantly increased radioiodine uptake in BCPAP cells in a dose- and time-dependent manner (Figure 1A,B). Increased radioiodine uptake was completely inhibited by KClO_4_, a specific inhibitor of NIS (Figure 1C).

### 3.2. Effects of GSK5182 on ERRγ and Iodide Handing Gene Expression in RAI-Refractory PTC Cells

**Figure 2:**
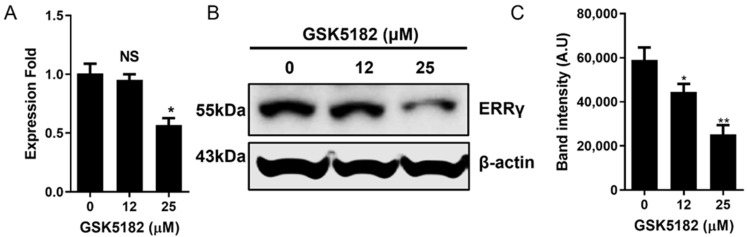
Effects of GSK5182 on ERRγ expression of BCPAP cells. Expression level of ERRγ (**A**) mRNA and (**B**) protein in GSK5182-treated cells. (**C**) Graph showing results of quantitative analysis of ERRγ protein levels by Image J. BCPAP cells were treated with GSK518 for 24 h, followed by collection of RNA and protein samples. *, *p* < 0.05. **, *p* < 0.005, NS, not significant. Data are the mean ± SD of three samples per group.

As shown in Figure 2A,B, GSK5182 decreased ERRγ expression in BCPAP cells at both the mRNA and protein levels. At the mRNA level, a significant decrease in *ERRγ* expression was observed at 25 μM, but not at 12 μM. A dose-dependent reduction in ERRγ protein levels was observed at both concentrations.

**Figure 3:**
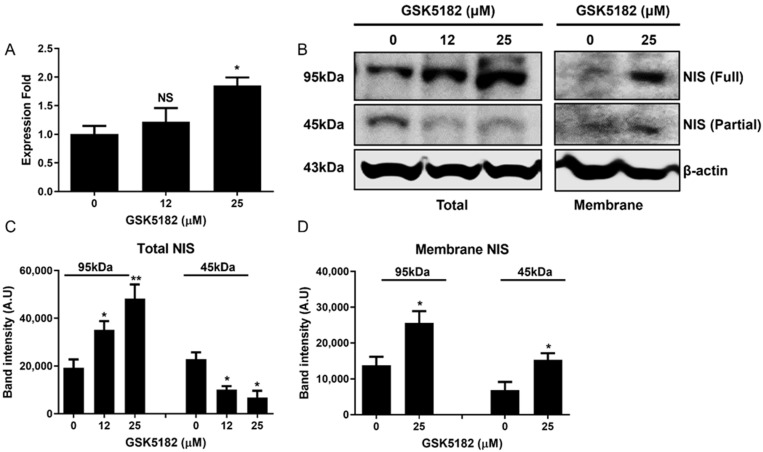
Effects of GSK5182 on NIS expression of BCPAP cells. Expression level of ERRγ (**A**) mRNA and (**B**) protein in GSK5182-treated cells. (**C**,**D**) Graph showing results of quantitative analysis of total and membrane NIS protein levels by Image J. BCPAP cells were treated with GSK5182 for 24 h, followed by collection of RNA and protein samples. *, *p* < 0.05. **, *p* < 0.005, NS, not significant. Data are the mean ± SD of three samples per group.

**Figure 4:**
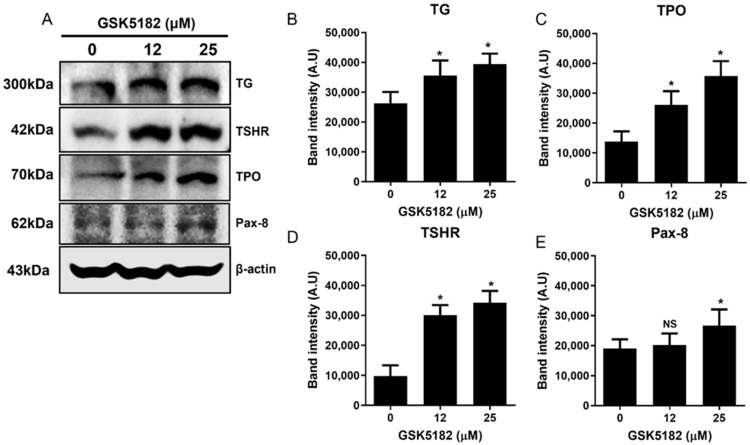
Effects of GSK5182 on iodide handing genes of BCPAP cells. (**A**) Expression level of TG, TSHR, TPO, and Pax-8 protein in GSK5182-treated cells. (**B**–**E**) Graph showing results of quantitative analysis of iodide metabolism-related genes by Image J. BCPAP cells were treated with GSK518 for 24 h, followed by collection of RNA and protein samples. *, *p* < 0.05. NS, not significant. Data are the mean ± SD of three samples per group.

In contrast to GSK5182-induced ERRγ expression, NIS mRNA expression increased in a dose-dependent manner (Figure 3A). An increase in NIS protein was found in GSK5182-treated cells but not in vehicle-treated cells (Figure 3B,C). Importantly, GSK5182 effectively induced a marked increase in membranous NIS fully and partially glycosylated protein (95 kDa and 50 kDa, respectively; Figure 3B,D). Western blotting revealed upregulation of iodide metabolism-related genes such as *TPO*, *TG*, *TSHR*, and *PAX-8* in GS5182-treated cells but not in vehicle-treated cells (Figure 4A–E).

### 3.3. Effects of Mitogen-Activated Protein (MAP) Kinase Signaling on GSK5182-Induced Radioiodine Uptake in RAI-Refractory PTC Cells

**Figure 5:**
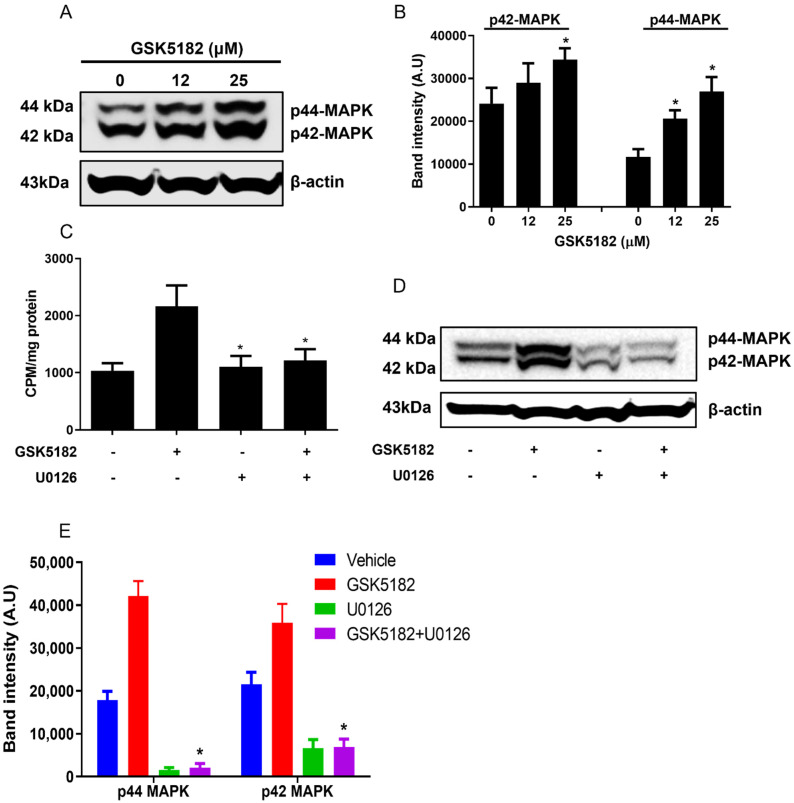
Effects of MAPK pathway on GSK5182-mediated iodine avidity in BCPAP cells. (**A**) Upregulation of pERK1/2 in GSK5182-treated cells. (**B**) Graph showing results of quantitative analysis of pERK1/2 levels by Image J. (**C**) Inhibition of iodide uptake in GSK5182-treated cells by U0126. (**D**) Reduction in pERK1/2 in GSK5182-treated cells by U0126. (**E**) Graph showing results of quantitative analysis of pERK1/2 levels by Image J. *, *p* < 0.05. NS, not significant. Data are the mean ± SD of three samples per group.

As depicted in Figure 5A,B, phosphorylated MAP kinase levels, such as p44 and p42 ERK (pERK1/2), were drastically increased in BCPAP cells by GSK5182, consistent with our previous findings [20].

Increased radioiodine uptake by GSK5182 was completely inhibited by U0126, a MEK-specific inhibitor (Figure 5C). Additionally, GSK5182-mediated upregulation of pERK1/2 was blocked to basal levels by U0126 treatment. U0126 alone did not lead to an increase in radioiodine avidity in BCPAP cells (Figure 5D,E).

### 3.4. Effects of GSK5182 on Glucose Metabolism in RAI-Refractory PTC Cells

**Figure 6:**
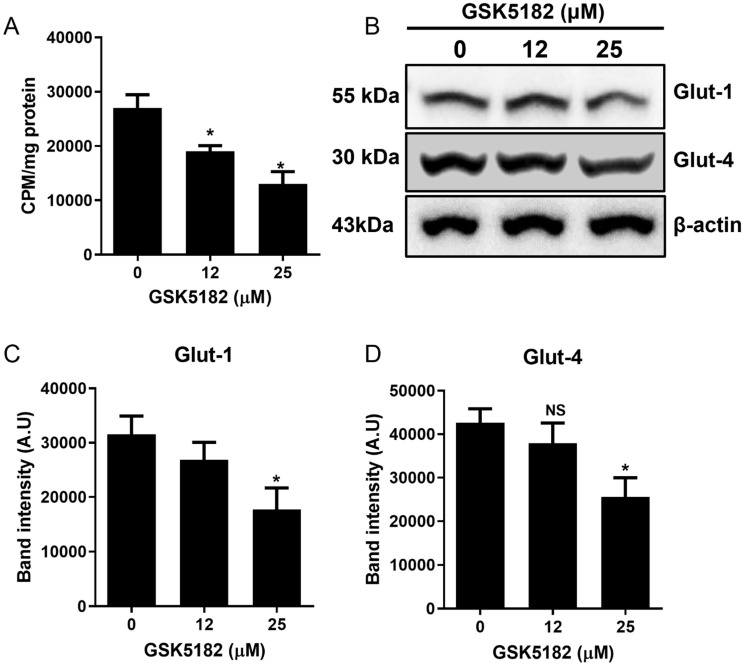
Effects of GSK5182 on glucose metabolism in BCPAP cells. (**A**) Change of ^18^F-FDG uptake in BCPAP cells by GSK5182. (**B**) Expression of glucose transporter 1 and 4 in GSK5182-treated BCPAP cells. (**C**,**D**) Graph showing results of quantitative analysis of Glut1/2 levels by Image J. *, *p* < 0.05. NS, not significant. Data are the mean ± SD of three samples per group.

GSK5182 significantly reduced ^18^F-FDG uptake in BCPAP cells in a dose-dependent manner (Figure 6A). Furthermore, the endogenous protein expression of glucose transporters like GLUT1 and GLUT4 decreased in GSK5182-treated cells but not in vehicle-treated cells (Figure 6B,D).

### 3.5. Restoration of Radioiodine Therapy Responsiveness in RAI-Refractory PTC Cells by GSK5182

**Figure 7:**
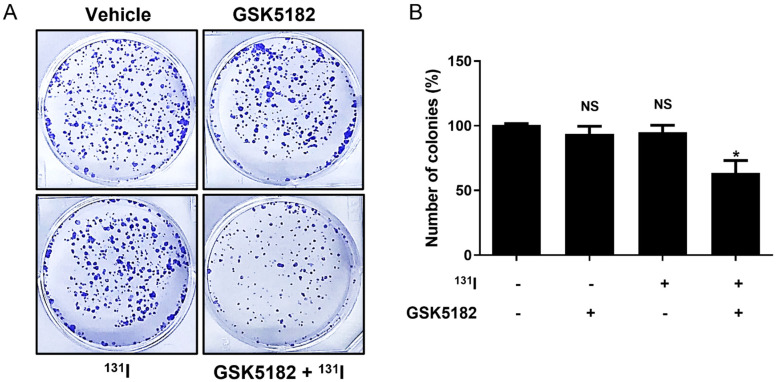
Enhanced cytotoxicity of radioiodine therapy by GSK5182 in BCPAP cells. (**A**) Photograph of crystal violet staining in BCPAP cells treated with GSK5182 or I-131 alone, and their combination. (**B**) Graph showing results of colony number in each well. *, *p* < 0.05. NS, not significant. Data are the mean ± SD of three samples per group.

Compared with vehicle-treated cells, ^131^I and GSK5182 showed no obvious cytotoxicity (Figure 7A,B). However, the combination of ^131^I and GSK5182 markedly increased cytotoxicity in RAI-refractory BCPAP cells. The relative colony-forming ability was 93.8 ± 5.8, 95.1 ± 5.2, and 63.5 ± 9.5% in the ^131^I-treated cells, GSK5182-treated cells, and ^131^I+GSK5182 cells, respectively.

## 4. Discussion

The incidence of thyroid cancer (TC) has increased drastically over the last few decades and is among the most rapidly increasing cancers globally. This increase is primarily due to the detection of many small tumors, mostly of the papillary subtype or PTC. PTC is the most common type of TC (approximately 80% of all TCs) known to affect humans, and its occurrence has steadily increased, threefold over the past 30 years [1,2,21,22,23]. Most PTCs are effectively treated by various therapeutic approaches, such as surgery, RAI therapy, tyrosine kinase inhibitors, TSH suppression, and their combination [24]. Among them, RAI is mainly used for PTC treatment owing to the well-known mechanism of iodide metabolism by its regulated genes, such as NIS, *TPO*, *TG*, and *TSHR*. It has been demonstrated that genetic aberrations, including BRAF, RAS, and RET/PTC rearrangements, induce development and dedifferentiation of differentiated TCs via activation of the mitogen-activated protein kinase (MAPK) and phosphoinositide 3-kinase (PI3K)/AKT signaling pathways, thereby inducing resistance to RAI therapy. Occasionally, PTC is dedifferentiated to become a more aggressive and lethal TC type with severe metastasis via various genetic mutations and abnormal occurrence of normal signaling pathways. Such PTCs do not respond to RAI therapy because these malignancies have lost their radioiodine avidity. Therefore, there is an urgent need to develop novel alternative therapeutic approaches.

It has been documented that RAI-refractory PTC with poor differentiation exhibits low NIS expression and radioiodine avidity [1,25]. First, to determine whether GSK5182 restored radioiodine avidity in RAI-refractory PTC, we used BCPAP cells that showed poor differentiation and low radioiodine uptake. Interestingly, GSK5182 increased radioiodine uptake in a dose- and time-dependent manner. Furthermore, we observed the complete inhibition of increased radioiodine uptake in GSK5182-treated BCPAP cells by KCLO_4_, an NIS-specific inhibitor. Based on these findings, we presume that an increased radioiodine uptake is linked to the status of GSK5182-mediated endogenous ERRγ. Immunoblotting with ERRγ-specific antibodies clearly revealed the presence of ERRγ protein in BCPAP cells and a drastic decrease in the endogenous ERRγ protein level in BCPAP cells by GSK5182. These findings suggest that the increase in radioiodine uptake results from enhanced NIS functional activity by the GSK5182-induced reduction in ERRγ.

Previous reports have shown the involvement of MAPK pathway proteins, such as pERK1/2, in the restoration of radioiodine uptake in anaplastic TC cells [20]. Consistently, we found that GSK5182 led to the activation of pERK1/2 in a dose-dependent manner in BCPAP cells. Inhibition of increased pERK1/2 using its specific inhibitor also resulted in the reduction of GSK5182-induced radioiodine uptake to basal levels. These data indicate the importance of MAPK kinase activation in the ERRγ-regulated increase in radioiodine avidity in RAI-refractory PTC cells.

Successful radioiodine therapy is dependent on the total amount of NIS protein and plasma membrane-localized NIS protein in the thyroid cells [1,26]. Poorly differentiated TC cells mostly show immature NIS protein (partially glycosylated NIS) at the total and membrane protein levels, which leads to poor uptake of radioiodine. Therefore, we examined the change in the total and plasma membrane NIS protein status by GSK5182. As expected, we observed partially glycosylated NIS in vehicle-treated BCPAP cells, with low expression in membrane NIS protein. However, GSK5182 markedly induced an increase in the total and membrane NIS protein in a fully glycosylated form. Although we cannot explain the mechanism by which total and membrane NIS protein are effectively increased, it may be linked to posttranslational mechanisms such as glycosylation and phosphorylation [27].

Poorly differentiated TC showed not only a downregulation of other iodide-regulating genes, which are important components of iodide metabolism, but also an increased glucose metabolism [25,28]. Another therapeutic approach to restore radioiodine avidity is the redifferentiation of RAI-refractory TC. In this study, we found that ERRγ modulation with GSK5182 induced an increase in iodide metabolism-related genes, and that GSK5182 effectively decreased glucose transporter and glucose uptake in BCPAP cells. These data suggest that GSK5182 provides a re-differentiation ability to RAI-refractory cells.

A drastic increase in radioiodine avidity in RAI-refractory BCPAP cells by GSK5182 could be expected to show potential cytotoxic effects. Our data show that GSK5182 enhanced the killing effect of I-131 on RAI-refractory BCPAP cells, but neither I-131 nor GSK5182 showed a cytotoxic effect in BCPAP cells. These findings suggest that the restoration of GSK5182-mediated radioiodine avidity induces sufficient cytotoxicity of I-131 in RAI-refractory BCPAP cells.

## 5. Conclusions

We have successfully demonstrated that functional modulation of the orphan nuclear receptor ERRγ is a reasonable therapeutic approach to overcome RAI resistance in RAI-refractory PTC cells. We have also found that the restoration of radioiodine avidity is closely related to ERRγ-modulated MAP kinase activation, thereby leading to an increased susceptibility to radioiodine therapy in RAI-resistant PTC cells. Moreover, our data indicate that GSK5182 exhibits unique characteristics that re-differentiate RAI-refractory PTC cells, by effectively restoring iodide metabolism-related genes and downregulating glucose metabolism. Further investigations are required to explore the detailed mechanism by which GSK5182 restores radioiodine avidity in RAI-refractory TC cells.

## Figures and Tables

**Table 1:** List of used antibodies for western blot analysis.

Primary Antibodies				Secondary Antibodies			
Name	Dilution Factor	Catalog Number	Manufacturer	Name	Dilution Factor	Catalog Number	Manufacturer
**NIS**	1:4000	MS-1653	Thermo Fisher	Anti-Mouse IgG, HRP-linked Antibody	1:8000	402B	PROMEGA
**TTF-1**	1:1000	SC-53136	Santa Cruz	Anti-Mouse IgG, HRP-linked Antibody	1:2000	402B	PROMEGA
**PAX-8**	1:1000	SC-81353	Santa Cruz	Anti-Mouse IgG, HRP-linked Antibody	1:2000	402B	PROMEGA
**Thyroperoxidase (TPO)**	1:1000	SC-58432	Santa Cruz	Anti-Mouse IgG, HRP-linked Antibody	1:2000	402B	PROMEGA
**TSH-receptor (TSHR)**	1:1000	SC-58432	Santa Cruz	Anti-Mouse IgG, HRP-linked Antibody	1:2000	402B	PROMEGA
**ERRγ**	1:1000	PP-H6812-00	R&D system	Anti-mouse IgG Isotype, HRP-linked Antibody	1:2000	402B	PROMEGA
**Phospho-p44/42 MAPK (Erk1/2)**	1:1000	4377	Cell Signaling	Anti-Rabbit IgG, HRP-linked Antibody	1:10,000	401B	PROMEGA
**GLUT-1**	1:1000	sc-377228	Santa Cruz	Anti-Mouse IgG, HRP-linked Antibody	1:10,000	401B	PROMEGA
**GLUT-4**	1:1000	ab33780	Abcam	Anti-Rabbit IgG, HRP-linked Antibody	1:10,000	401B	PROMEGA
**β-actin**	1:5000	ab8227	Abcam	Anti-Rabbit HRP-linked Antibody	1:000	401B	PROMEGA

## Data Availability

Data will be made available on a reasonable request.

## Supplementary Materials

The following supporting information can be downloaded at: https://www.mdpi.com/article/10.3390/cells12030470/s1.
