# Supplementary material for: An Inverse Agonist of Estrogen-Related Receptor Gamma, GSK5182, Enhances Na+/I− Symporter Function in Radioiodine-Refractory Papillary Thyroid Cancer Cells
Source: Cells. 2023 Feb 1;12(3):470. doi: 10.3390/cells12030470 (PMC9914548; doi:10.3390/cells12030470)
Supplement: Supplementary file 1 [file cells-12-00470-s001.zip › cells-2099289-supplementary.pdf]

An Inverse Agonist of Estrogen-Related Receptor Gamma, GSK5182, Enhances Na^+/I^- Symporter Function in Radioiodine-Refractory Papillary Thyroid Cancer Cells

Thoudam Debraj Singh [1,*,†], Jae-Eon Lee [2,†], Kwang-Hee Son [2], Bo-Ra Lee [2], Sang-Kyoon Kim [2], Deepak Gulwani [1], Vijaya Sarangthem [3] and Yong-Hyun Jeon [2,*]

1 Department of Medical Oncology Lab, All India Institute of Medical Sciences (AIIMS), 110029 New Delhi, India; d.gulwani@gmail.com

2 Preclincial Research Center (PRL), Daegu-Gyeongbuk Medical Innovation Foundation (K-MEDI hub), 41061 Daegu, Republic of Korea; koof12@kmedihub.re.kr (J.-E.L.); sonkh33@kmedihub.re.kr (K.-H.S.);

damsmom68@kmedihub.re.kr (B.-R.L.); ksk1420@kmedihub.re.kr (S.-K.K.)

3 Department of Pathology, All India Institute of Medical Sciences (AIIMS), 110029 New Delhi, India; devi1703@gmail.com

* Correspondence: debraj.thoudam@gmail.com (T.D.S.); jeon9014@gmail.com (Y.-H.J.)

† The first two authors contributed to this study equally.

MATERIALS AND METHODS

Cells, chemicals, and antibodies

Papillary thyroid cancer cell line, BCPAP cells, was purchased from Deutsche Sammlung von Mikroorganismen und Zellkulturen. BCPAP cell lines were maintained in DMEM high supplemented with 10% FBS, 1% antibiotic-antimycotic (Hyclone) at 37 ºC in a 5% CO_2 atmosphere. Primary mouse monoclonal human NIS–specific antibody (Thermo Scientific, IL, USA), ERRγ (R&D, MN, USA), phospho-MAPK-p42/p44 (Cell Signaling, MA, USA), GLUT-4 and β-actin antibody (Abcam, MA, USA), thyroidperoxidase (TPO), thyroid stimulating hormone receptor (TSHR), thyroglobulin (TG), PAX-8, GLUT-1 from Santa Cruz biotechnology Inc.

Radioiodine uptake Reporter gene assay

For the I-125 uptake assay, BCPAP cells were plated in 24-well plates for 24h and then treated with GSK5182 (synthesized by Daegu-Gyeongbuk Medical Innovation Foundation (DGMIF, Daegu, Korea and prepared as a 50mM stock solution in DMSO and stored at -80°C) for 24 h. After aspirating drug-containing medium, cells were washed with 1mL HBSS (Hank's balanced salt solution) and incubated with 500µL of (HBSS) containing 0.5% bovine serum albumin (bHBSS), 3.7 kBq carrier-free ^{125}I (Perkin-Elmer) and 10µmol/L sodium iodide (specific activity of 740MBq/mmol) at 37°C for 30 min. The cells were then washed twice with ice-cold bHBSS and were lysed with 500µl of 2% sodium dodecyl sulfate (SDS). The radioactivity was measured using a gamma counter (Packard Cobra II gamma-counter (PerkinElmer, MA). The radioactivity of the cells was normalized using total protein concentrations determined by a BCA kit (Pierce Protein Biology). To inhibit the iodide uptake, cells were pre-incubated with 300 µM $KClO_4$ (as a specific inhibitor for NIS) for 30

min and then treated with ^{125}I as described above.

^{18}F-FDG Uptake Assay

The 1x10^5 BCPAP cells were seeded in each well of a 24-well plate then treated with GSK5182 for 24 h. After aspirating drug-containing medium, cells were washed with 1mL HBSS (Hank's balanced salt solution) and incubated with 500μL of HBSS containing 0.5% bovine serum albumin (bHBSS), 74 kBq of 18F-FDG per milliliter for 30 min at 37℃. The collection of cell lysate and the measurement of radioactivity and the protein contents of the supernatants were also performed as described for the ^{125}I uptake study.

Clonogenic Assay

BCPAP cells were plated into 6-well plates and left for 48 h. After treatment with 25 μM GSK5182 for 24h, drug-containing medium was discarded and cells were washed twice with PBS. The medium was then replaced with DMEM in the presence or absence of 50μCi ^{131}I (KIRAMS, Korea) for 6 h. Cells were washed with cold bHBSS and left in regular culture medium for the time corresponding to six doublings. Finally, cells were fixed in 4% Paraformaldehyde (PFA) solution and stained with 0.05% crystal violet. Control and ^{131}I treated colonies with over than 50 cells were counted.

Western Blot

The BCPAP cells were treated with or without GSK5182 for 24h. It were washed twice with cold PBS and lysed with RIPA buffer containing complete protease inhibitor cocktail (Roche). In case of plasma membrane protein for NIS, samples were prepared with protein biotinylation kit (EZ-Link™Sulfo-NHS-Biotin, Thermo Scientific) according to

manufacturer's instruction. Briefly, either untreated- or treated-cells were washed twice with ice-cold PBS/CM (PBS containing 0.1 mM calcium chloride and 1 mM magnesium chloride, pH 7.3) and incubated with EZ link NHS-Sulfo-SS-biotin (1 mg/mL) in PBS/CM for 30 min at 4 ºC. The reaction was quenched by 2 washes with cold 100 mM glycine in PBS/CM and further incubation with 100 mM glycine in PBS/CM at 4 ºC for 20 min. Cells were then quickly washed two times with PBS/CM before lysis with RIPA buffer containing protease inhibitors cocktail and phosphatase inhibitors (Roche) for 1 h at 4 °C with constant shaking. Lysates were centrifuged at 16,000g for 30 min at 4 ºC. A portion of the supernatant was used for total cell protein immunoblots. The remaining sample was used to obtain membrane protein by incubation with 100 µL streptavidin beads (Thermo Scientific) for 1 h at room temperature. Beads were washed 3 times with RIPA buffer, and bound proteins were eluted with 50 µL of Laemmli buffer (62.5 M Tris, pH 6.8; 20% glycerol; 2% SDS; 5% b-mercaptoethanol; and 0.01% bromphenol blue) for 30 min at room temperature. Equal amounts of total and biotinylated plasma membrane protein were loaded in each lane and resolved by 4-12% gradient Bis-Tris gel (Invitrogen). Proteins were transferred to 0.2-µm PVDF membrane (Invitrogen). Membranes were incubated overnight at 4°C with primary mouse monoclonal human NIS–specific antibody (dilution, 1:1000, Thermo Scientific, Catalog#: MS-1653-P1, Clone: FP5A), followed by incubation with HRP-conjugated secondary antibody at room temperature. ECL-Plus (Amersham Pharmacia) was used to detect peroxidase activity according to the manufacturer's protocol.

Similarly, for other protein also, equal amounts of protein were loaded in each lane and resolved by 4-12% gradient Bis-Tris gel (Invitrogen). Proteins were transferred to 0.2-µm PVDF membrane (Invitrogen). Membranes were incubated overnight at 4°C with primary antibodies and then incubated with the appropriate HRP-conjugated secondary antibody at room temperature. ECL-Plus was used to detect peroxidase activity according to the

manufacturer's protocol. Band densities were determined by ImageJ software.

Quantitative RT-PCR.

Total RNA was extracted using Trizol (Invitrogen, Carlsbad, CA) and reverse transcription reaction was performed on 2 μg of total RNA using RevertAid First Strand cDNA Synthesis Kit (Thermo Scientific, Pittsburgh, PA). Quantitative RT-PCR was carried out with SYBR Green PCR master mix (Applied Biosystems, Foster City, CA) using a ViiA 7 Real-Time PCR System instrument (Applied Biosystems) with the following primer sets: ERRγ (forward, 5'- CAG ACG CCA GTG GGA GCT A -3'; reverse, 5'- TGG CGA GTC AAG TCC GTT CT - 3'), NIS (forward, 5'- TCT AAC CGA TGC TCA CCT CTT CTG -3'; reverse, 5'- AGA TGA TGG CAC CTC CTT GAA CC -3'), and acidic ribosomal protein 36B4 (forward, 5'- CCA CGC TGC TGA ACA TGC T -3'; reverse, 5'- TCG AAC ACC TGC TGG ATG AC -3'). The target genes were normalized to the endogenous reference gene 36B4, and relative mRNA expression levels were calculated in the test and control samples.

Statistical analysis

All data are expressed as the mean ± standard deviation (SD) from at least three representative experiments, and statistical significance was determined using an unpaired Student's test. p-values of < 0.05 were considered statistically significant.
